# Which Assisted Living Communities Provide Hospice?

**DOI:** 10.1093/geroni/igaa057.2355

**Published:** 2020-12-16

**Authors:** Lindsay Prizer, Sheryl Zimmerman, Christopher Wretman, John Preisser, Kali Thomas, Philip Sloane

**Affiliations:** 1 Emory University School of Medicine, Atlanta, Georgia, United States; 2 University of North Carolina at Chapel Hill, Chapel Hill, North Carolina, United States; 3 Brown University, Providence, Rhode Island, United States

## Abstract

Assisted living (AL) communities have become a common site for end-of-life and hospice care. However, AL is highly variable, meaning that hospice use is likely to be variable as well. This study explored the association between AL community characteristics and their residents’ use of hospice. A stratified random sample of 250 AL communities in seven states was recruited. Community-level data were obtained from interviews with AL administrators, and resident-level case-mix data were abstracted from charts. Survey-weighted regressions examined the relationship between community characteristics and hospice use. Having residents on hospice was associated with being for-profit (86% vs. 51%), larger (48 vs. 31 beds), newer (16 vs. 37 years), having weekly primary care provider visits (44% vs. 26%), having more residents with dementia (50% vs. 35%) and fewer on Medicaid (4% vs. 11%), and having more lenient discharge policies. Data suggest there may be some disparity in hospice provision in AL.

